# Multiple electrolytes imbalances in a patient with inflammatory bowel disease associated with vitamin D deficiency: a case report

**DOI:** 10.1186/s13256-023-04302-4

**Published:** 2024-01-22

**Authors:** Yumiko Nakamura, Yuichiro Kawai, Sumiko Nagoshi, Tomonari Ogawa, Hajime Hasegawa

**Affiliations:** 1 Department of Nephrology and Hypertension, Saitama Medical Center, Saitama Medical University, 1981 Kamoda, Kawagoe, Saitama 350-8550 Japan; 2 Department of Gastroenterology and Hepatology, Saitama Medical Center, Saitama Medical University, 1981 Kamoda, Kawagoe, Saitama 350-8550 Japan

**Keywords:** Hypocalcemia, Hypophosphatemia, Hypokalemia, Hypomagnesemia, Parathyroid hormone, Crohn’s disease, Entero- Behçet’s disease

## Abstract

**Background:**

Inflammatory bowel disease involves chronic inflammation and ulceration, primarily Crohn’s disease and ulcerative colitis. The prevalence of inflammatory bowel disease is rising in industrialized countries. We describe the case of a patient with inflammatory bowel disease and multiple electrolyte disturbances that emphasize the link between a vitamin D deficiency and electrolyte imbalances.

**Case:**

An 86‐year‐old Japanese man with severe hypocalcemia, hypophosphatemia, hypokalemia, and hypomagnesemia was referred to the gastroenterology and hepatology department our university hospital for severe diarrhea and abdominal pain. Based on clinical symptoms and biochemical and endoscopic findings, Crohn’s disease, intestinal Behçet’s disease, and intestinal tuberculosis were considered as differential diagnoses, but a final diagnosis was not reached. Prednisolone, azathioprine, and metronidazole were administered, and no apparent electrolyte abnormality was observed at the patient’s admission to our hospital. On the 80th hospital day, marked hypocalcemia, hypophosphatemia, hypokalemia, and hypomagnesemia were noted and prolonged, despite daily supplementation with Ca and inorganic P. At his consultation with our department, we observed decreased fractional excretion of Ca, tubular reabsorption of phosphate, fractional excretion of K, and fractional excretion of Mg, suggesting the depletion of vitamin D and extrarenal wasting of K and Mg. The patient’s serum Ca and inorganic P were quickly elevated in response to treatment with an active form of vitamin D, and his serum levels of K and Mg were restored to the normal range by an intravenous administration of K and Mg. A vitamin D deficiency is not rare in inflammatory bowel disease and is caused primarily by the decreased intestinal absorption of vitamin D. In the management of electrolyte imbalances in patients with inflammatory bowel disease, clinicians must consider the possible development of vitamin D deficiency-related disorders.

**Conclusion:**

Vitamin D deficiency in entero-Behçet’s disease leads to severe hypocalcemia and hypophosphatemia, highlighting the importance of awareness in management.

## Background

Inflammatory bowel disease (IBD) is characterized by chronic inflammation and ulceration. Crohn’s disease and ulcerative colitis are typical forms of IBD; other intestinal disorders showing chronic inflammation and ulceration as principal impairments, such as entero-Behçet’s disease, should also be included in this disease category. The principal clinical signs of IBD include chronic and severe diarrhea, abdominal pain, body weight loss, and prolonged general fatigue. The prevalence of IBD is rapidly increasing in industrialized countries [1], and IBD cases are not rare in the field of nephrology. IBD commonly occurs in the ileocecal region and may cause malnutrition and malabsorption, as well as various electrolyte abnormalities. We treated a patient with IBD with various electrolyte disturbances. We report this case to highlight the need for clinicians to be aware that vitamin D deficiency can be caused by multiple electrolyte disturbances.

## Case presentation

We report the case of an 86‐year‐old Japanese man. Three months prior to his admission, he was diagnosed with ulcerative colitis by a physician at a clinic and was receiving prednisolone (15 mg/day) and azathioprine (75 mg/day). He was referred to the department of gastroenterology and hepatology at our university hospital due to an exacerbation of symptoms, including diarrhea and anal pain. At the time of his consultation with our gastroenterology department, no electrolyte abnormalities were observed; a sodium (Na) of 135 mEq/L and a potassium (K) of 4.3 mEq/L were recorded.

The patient’s bowel disorder was carefully examined by endoscopic observation and a colon biopsy. As candidates for the final diagnosis, severe ulcerative colitis, entero-Behçet’s disease, and ileocecal tuberculosis were considered. However, achieving the accurate and final differential diagnosis was difficult, and metronidazole (500 mg/day) and budesonide (9 mg/day) were additionally administered. Intestinal tuberculosis could not be entirely excluded, and thus, the combination of isoniazid (300 mg/day) and pyridoxal phosphate (30 mg/day) was started. Verapamil (80 mg/day) and cibenzoline succinate (150 mg/day) had been administered by the patient’s clinic physician for his arrhythmias. The patient’s other oral medications included esomeprazole (20 mg/day), pitavastatin (2 mg/day), sulfamethoxazole–trimethoprim (1 g/day), and alendronate (35 mg/week).

On the 80th hospitalized day, a decrease in the patient’s serum concentrations of K, calcium (Ca), inorganic phosphorus (iP), and magnesium (Mg) emerged and showed a worsening trend. The administration of sodium dihydrogen phosphate (1200 mg/day) as a phosphate supplementation and calcium l-aspartate (1200 mg/day) as a Ca supplementation was started from day 85. However, the multiple electrolyte disturbances worsened, and hypokalemia (2.3 mEq/L), hypocalcemia (7.3 mg/dL), and hypomagnesemia (0.9 mg/dL) were present on day 95, resulting in the patient’s consultation to our department. According to his medical history, he had experienced appendicitis at the age of 20 years, and he experienced hypertension and arrhythmia of unknown onset after surgery for transverse colon cancer at the age of 74 years.

The physical examination at the time of the patient’s consultation to our department showed significant emaciation: height 165 cm, weight 47.5 kg, and body mass index 17.5. His body temperature was 36.7 °C, blood pressure was 105/65 mmHg, and pulse rate was 77 beats/min. Muddy stools persisted from the time of admission. The patient’s hospital diet at his consultation in the nephrology department offered 1600 kcal/day and included 3106 mg/day of Na, 2220 mg/day of K, 237 mg/day of Mg, and 992 mg/day of iP.

The results of the laboratory tests at the time of consultation to our department are summarized in Table 1. In addition to multiple dyselectrolytemias, we observed metabolic alkalosis, proteinuria, and a remarkable elevation of the *N*-acetylglucosaminidase (NAG)-to-Cr ratio (NAG index) indicating renal tubulointerstitial impairment. A urine biochemical analysis showed decreased fractional excretions of K, Mg, and Ca. Conversely, the % tubular reabsorption of phosphate (%TRP) showed an increased iP excretion.

**Table 1:** The patient’s laboratory results at the time of consultation to our department

Blood biochemistry		Blood gas analysis	
Albumin, g/dL	2.2	pH	7.511
AST, IU/L	29	pO_2_, mmHg	22.0
ALT, IU/L	14	pCO_2_, mmHg	45.2
LDH, IU/L	338	HCO_3_^−^, mmol/L	35.3
Total bilirubin, mg/dL	0.3	Urinalysis	
BUN, mg/dL	14	Specific gravity	1.017
Cr, mg/dL	0.47	RBC count, per HPF	1–4
eGFR, mL/min	123.4	WBC count	1–4
Na, mEq/L	142	pH	7.5
Cl, mEq/L	97	Protein by dip stick	+ 1
K, mEq/L	2.3	Sugar by dip stick	±
cCa, mg/dL	5.5	Urine biochemistry	
i P, mg/dL	1.0	Cr, mg/dL	50.6
Mg, mEq/L	0.9	Protein, g/gCr	0.41
Blood sugar, mg/dL	71	NAG index, IU/gCr	28.2
HbA1c, %	5.6	FENa, %	0.81
Whole PTH, pg/mL	132.8	FEK, %	1.75
Cortisol, µg/dL	6.81	FEMg, %	2.06
Plasma aldosterone, pg/mL	10.0	FECa, %	0.30
Plasma renin activity, ng/mL/h	0.5	%TRP, %	39.1
Blood cell count			
RBC, × 10^4^ per µL	310		
Hemoglobin, g/dL	8.1		
Hct, %	25.1		
Plate, × 10^4^ per µL	15.5		

*AST* aspartate transaminase, *ALT* alanine aminotransferase, *LDH* lactate dehydrogenase, *BUN* blood urea nitrogen, *cCa* corrected Ca, *iP* inorganic P, *HPF* high power field, *NAG* *N*-acetyl-β-d-glucosaminidase, *FENa, K, Mg, Ca* fractional excretion of Na, K, Mg, Ca

Based on the findings of a decreased renal excretion of Ca and an increased excretion of iP, we suspected a decreased intestinal absorption of vitamin D (VD), and we then measured the patient’s 25-hydroxy (OH) VD concentration. We observed a decrease in 25-OH VD (10 ng/mL, normal range ≥ 21 ng/mL) and an increase in the whole form of parathyroid hormone (PTH). Renin and the aldosterone system were also suppressed. Because a decrease in VD was demonstrated, we began the administration of vitamin-D3, the active form of VD, with 0.25 μg/day of 1,25-vitamin D3, in addition to supplementation of iP and Ca with 930 mg/day of sodium phosphate and 400 mg/day calcium chloride (CaCl2) from day 97.

**Fig. 1:**
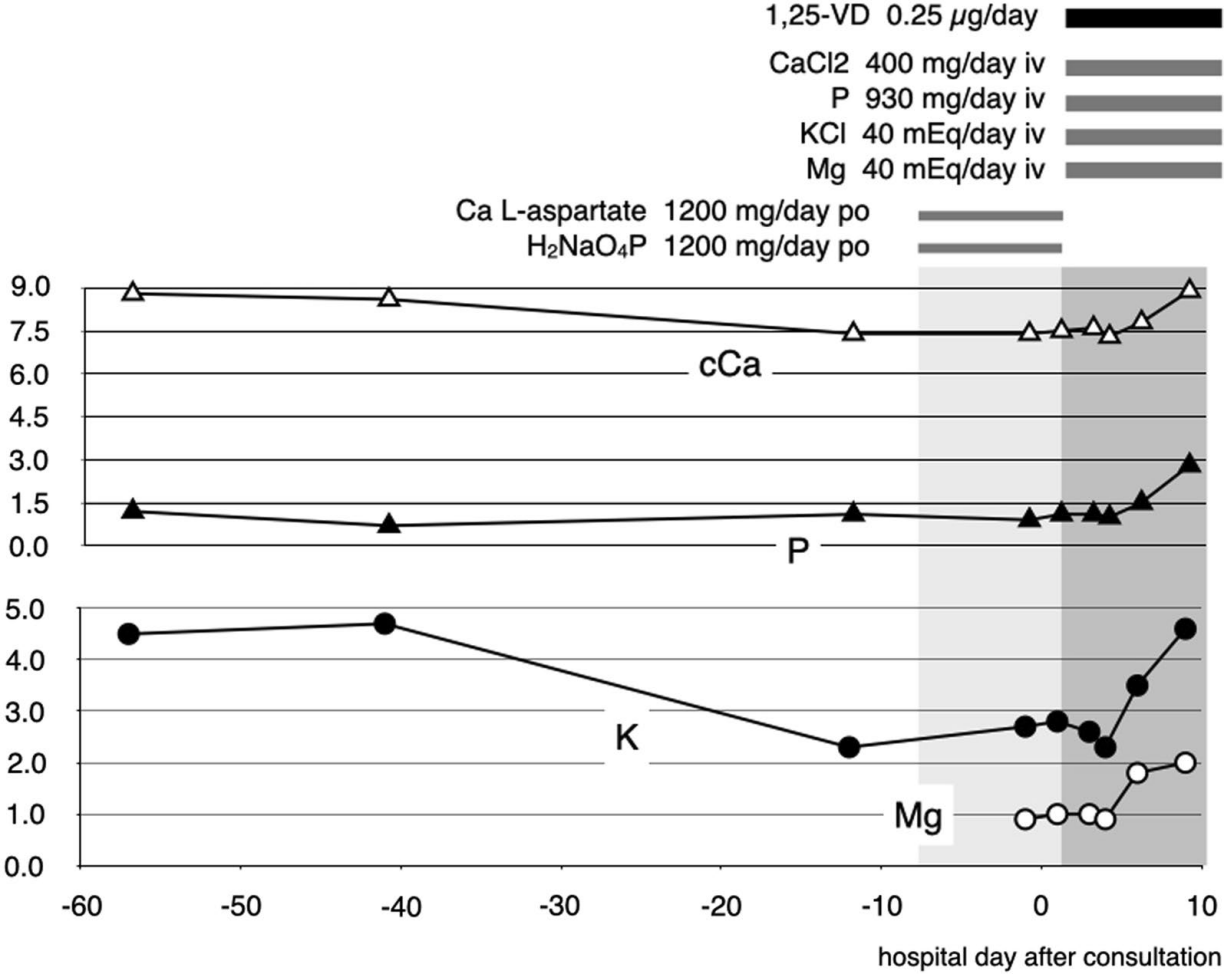
The course of treatment and trends in recovery of various electrolytes

**Fig. 2:**
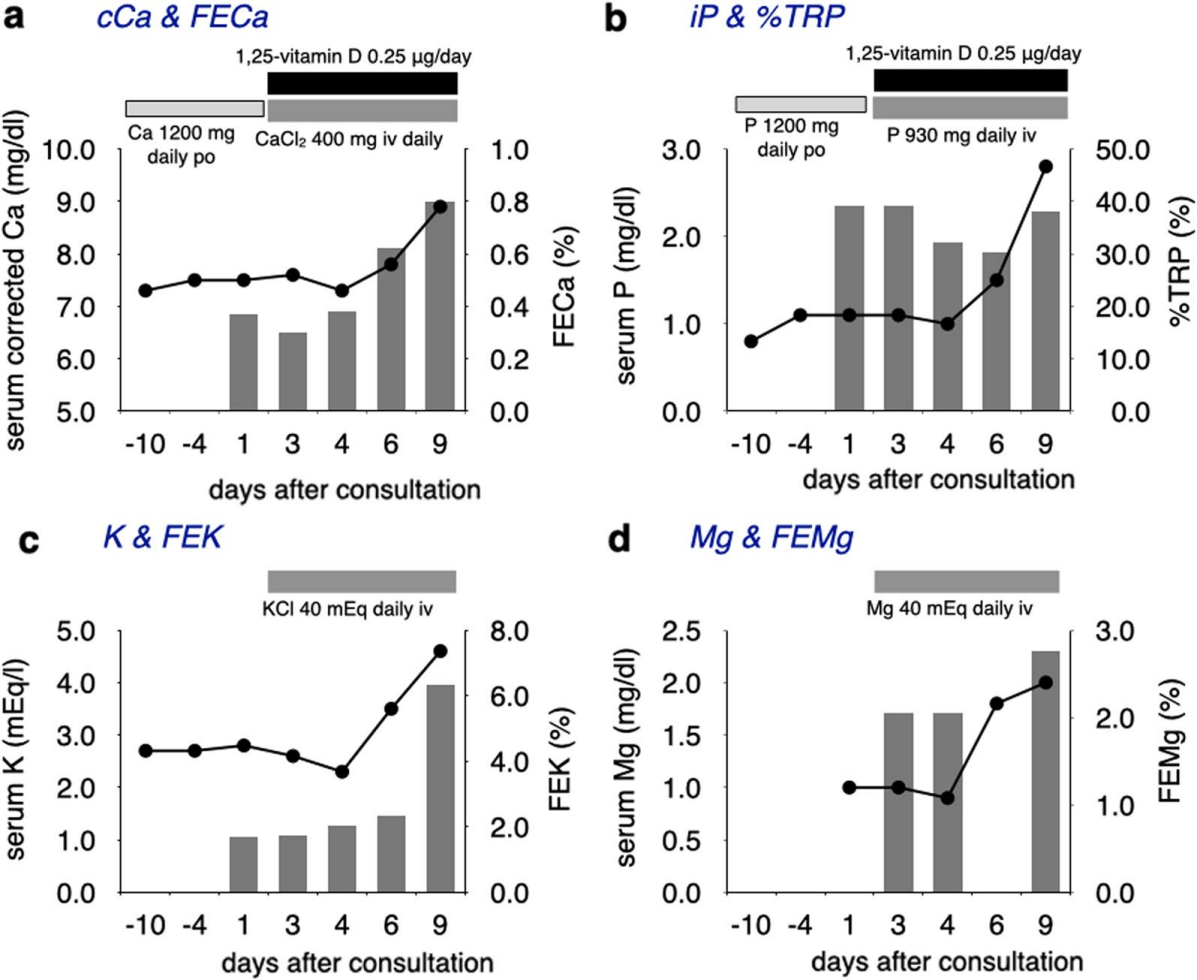
The laboratory data and urinary excretion rates of each electrolyte until the values normalize

The patient’s hypocalcemia and hypophosphatemia were quickly ameliorated after the introduction of VD supplementation, and both the Ca and iP concentrations were normalized at 1 week after the start of the VD supplementation (day 104). Supplementation with K (40 mEq/day) by intravenous potassium chloride (KCL) administration and Mg (20 mEq/day) by magnesium sulfate (MgSO4) were started from day 97, and on day 104, the patient’s K and Mg values were in the normal ranges (Fig. 1). Figure 2 shows the serum and urinary excretion rates of each electrolyte until the values normalize.

## Discussion and conclusion

Our patient had IBD associated with various electrolyte abnormalities, and we speculated that his dyselectrolytemia was composed of two groups of diseases, that is, hypocalcemia/hypophosphatemia and hypokalemia/hypomagnesemia.

We first discuss hypocalcemia and hypophosphatemia. Vitamin D is closely involved in the metabolism of Ca and iP and is delivered to the body in two ways: oral intake as a dietary nutrient and biosynthesis by ultraviolet radiation in the skin [2, 3]. Vitamin D incorporated into chylomicrons is metabolized in the liver to change to 25-(OH)VD_3_ and then metabolized to 1,25-(OH)_2_ VD in the kidneys, as the active form of VD showing bioactivity [3].

The active form of VD promotes Ca and iP resorption from the small intestine, resulting in increased Ca and iP levels in the blood [3]. The absorption of VD is generally lowered in gastrointestinal diseases [4–6]. Particularly, IBD in which the lesion occurs in the terminal ileum, such as Crohn’s disease and entero-Behçet’s disease, may reduce the absorption of VD, because bile acid diarrhea often occurs. Several factors are involved in the development of VD deficiency, including impaired absorption of nutrients and bile-salt malabsorption, restricted dietary intake, and limited exposure to sunlight because of long-time hospitalization [7]. Indeed, our patient developed the disturbance of multiple electrolytes associated with VD deficiency after more than 2 months of hospitalization. This long-term limited exposure to sunlight might be also involved in the development of VD deficiency.

VD deficiency commonly occurs in patients with IBD with a prevalence of approximately 30–40% [8–10]. A study of factors affecting the plasma concentration of 25-(OH)VD_3_ revealed that most the powerful predictor variable is not the amount of VD intake but the amount of fat intake [11]. Since restricting fat intake is a common dietary therapy for patients with IBD, a reduction in fat intake may have played a part in the development of VD deficiency in this case as well.

Despite marked hypophosphatemia, our patient’s %TRP was markedly lower and increased renal excretion of P appeared to be the principal cause of marked hypophosphatemia. The initial level of PTH was increasing, which may have largely contributed to the decrease in the rate of renal P reabsorption. Increased intestinal P absorption seemed to be the main reason for the increase in the serum P level after the initiation of VD supplementation, while the P reabsorption rate in the kidney remained low. Although it is possible that the high PTH level was prolonged, the detailed mechanism is unclear because no subsequent PTH or fibroblast growth factor 23 (FGF23) was measured in this case.

Our patient’s hypokalemia and hypomagnesemia are of note. Generally, the mechanism of the development of hypokalemia is roughly divided into three categories: (1) the facilitation of K migration into the cells or the inhibition of K release to the extracellular space, (2) the facilitation of K excretion from the kidneys, and (3) extrarenal K loss [11].

In our patient, we speculate that the likelihood of category 1 is low, because a drug that facilitates K transfer into the cells was not used, and his blood sugar level was also normal. In addition, he showed consistently low urine K excretion during his clinical course, indicating that the principal cause of his hypokalemia was not an increase in the renal K excretion.

These findings suggest that extrarenal K wasting may be one of the causes of our patient’s hypokalemia, and that the presence of IBD may be associated with increased fecal K excretions. The fecal K level was not measured in our patient, but a K loss associated with severe diarrhea is not rarely encountered in the clinical medicine. In addition, the patient’s massive intestinal fluid loss also led to the loss of chloride as well as K, which may have been a cause of his hypochloremia.

Our patient’s severe hypomagnesemia also merits discussion. The primary site of Mg absorption in the gastrointestinal tract is the small intestine, particularly the distal small intestine [12], and the primary cause of hypomagnesemia is thought to be decreased absorption from the intestinal tract. Indeed, hypomagnesemia is often seen in patients with diseases presenting with a malabsorption of nutrients, and a decrease in the absorbing area of the intestine might be presumed as a cause of the decreased Mg absorption in patients with IBD. A vitamin D deficiency, which facilitates Mg resorption, may also contribute in part to the development of hypomagnesemia.

It has been reported that K and Mg deficiencies are closely related; 40–60% of patients with hypomagnesemia show hypokalemia [13], and the conditions of 50% of patients with hypokalemia are associated with hypomagnesemia [14]. In addition, the presence of a Mg deficiency accelerates the excretion of K from the renal collecting ducts, and hypokalemia is likely to be refractory to treatment. This is because the activity of renal outer medullary potassium (ROMK) channels, which play a role in aldosterone-sensitive K excretion from the renal collecting ducts, is negatively controlled by the binding of Mg on the channel protein, and Mg deficiency attenuates this regulation [14]. In our patient, the losses of K and Mg from the gastrointestinal tract may have contributed to the sustained settings of hypomagnesemia and hypokalemia, respectively. In the management of patients showing dyselectrolytemia, clinicians should be aware that the presence of infection and/or chronic disease can worsen the prognosis of any electrolyte imbalance [15].

In summary, we treated a patient who exhibited multiple electrolyte abnormalities associated with IBD. In his case, the vitamin D deficiency resulting from malabsorption in the terminal ileum would be principally involved in the cause of his hypocalcemia and hypophosphatemia. In the management of entero-Behçet’s disease, and presumably inflammatory bowel syndrome diseases such as Crohn’s disease, clinicians should be aware that malabsorption of vitamin D may cause severe hypocalcemia and hypophosphatemia.

## Data Availability

Not applicable.

## Abbreviations

Ca: Calcium
CaCl2: Calcium chloride
IBD: Inflammatory bowel disease
iP: Inorganic phosphorus
K: Potassium
Mg: Magnesium
MgSO4: Magnesium sulfate
Na: Sodium
NAG: *N*-acetylglucosaminidase
PTH: Parathyroid hormone
VD: Vitamin D
%TRP: Tubular reabsorption of phosphate
